# Raising Children on a Vegan Diet: Parents’ Opinion on Problems in Everyday Life

**DOI:** 10.3390/nu13061796

**Published:** 2021-05-25

**Authors:** Daisy Bivi, Teresa Di Chio, Francesca Geri, Riccardo Morganti, Silvia Goggi, Luciana Baroni, Maria Gloria Mumolo, Nicola de Bortoli, Diego Giampietro Peroni, Santino Marchi, Massimo Bellini

**Affiliations:** 1 Gastrointestinal Unit, Department of Translational Sciences and New Technology in Medicine and Surgery, University of Pisa, 56124 Pisa, Italy; bividaisy@gmail.com (D.B.); f.geri91@gmail.com (F.G.); g.mumolo@int.med.unipi.it (M.G.M.); nicola.debortoli@unipi.it (N.d.B.); s.marchi@med.unipi.it (S.M.); massimo.bellini@unipi.it (M.B.); 2 Pediatric Institute of Southern Switzerland, Ospedale Regionale di Bellinzona e Valli, Via Ospedale 12, 6500 Bellinzona, Switzerland; 3 Section of Statistics, University Hospital of Pisa, 56100 Pisa, Italy; r.morganti@ao-pisa.toscana.it; 4 Scientific Society for Vegetarian Nutrition, Scientific Committee, Via Verdi 10/9, 30171 Mestre (VE), Italy; silvia.goggi@sanpiox.humanitas.it (S.G.); luciana.baroni@scienzavegetariana.it (L.B.); 5 Department of Clinical and Experimental Medicine, Section of Pediatrics, University of Pisa, 56126 Pisa, Italy; diego.peroni@unipi.it

**Keywords:** vegan diet, vegetarian diets, nutrition, complementary feeding, weaning, children, infants

## Abstract

A growing number of Italian families are adopting a vegan diet (VD) for their offspring from infancy for various reasons, with health benefits and ethics being the most common reasons. Barriers to effective communication with primary care pediatricians (PCPs) are perceived by many parents and, depending on the actors involved and the environment, a VD may affect social interactions in everyday life. A national cross-sectional survey was conducted between July and September 2020. Parents of children following a VD completed an online questionnaire. Data from 176 Italian parents were collected. About 72% (71.8%) of the children included in this study had been on a VD since weaning. Parents did not inform their primary care pediatricians (PCP) about the VD in 36.2% of the cases. In 70.8% of the cases, PCPs were perceived as skeptical or against a VD. About 70% (71.2%) of the parents relied on medical dietitians, and 28.2% on nutritionists/dietitians for dietary counseling. Parents administered an individual B12 supplement in 87.2% of the cases. To the best of our knowledge, this survey is the first which explores the relationship between vegan parents and their PCPs, the parental management of their children’s diet and problems regarding the implementation of a VD in everyday life.

## 1. Introduction

**Figure 1:**
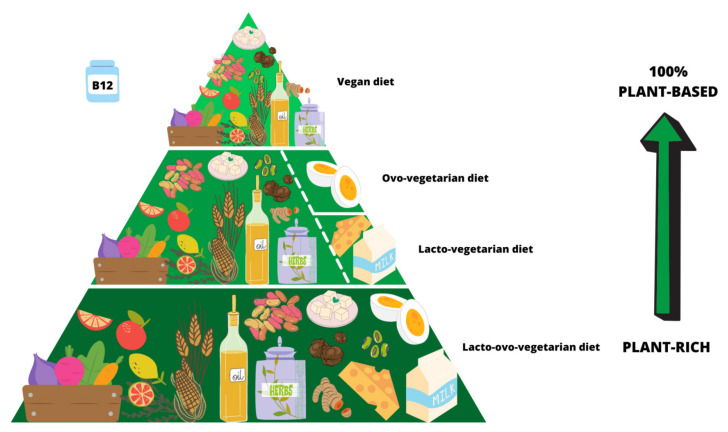
Vegetarian diets: from plant-rich eating patterns (lacto-ovo-vegetarian, lacto-vegetarian, ovo-vegetarian diets) to 100% plant-based (vegan diet). There are no reliable sources of vitamin B12 in plants, so supplementation is crucial.

According to the report released by Eurispes in May 2021 [1], people following a vegan diet (VD) are estimated to be 2.4% of the total population in Italy. Among those who consume no food that comes from animals (meat, eggs, dairy products), there is a growing number of mothers and fathers adopting a VD for their offspring. A VD, also defined as a 100% plant-based diet, is the strictest form of vegetarianism as it avoids all animal-derived foods. Lacto-ovo-vegetarian diets are plant-rich: they exclude meat but include dairy and/or eggs (Figure 1).

The more restricted the diet, the greater the risk of deficiencies, but a VD should not be intended as a calorie- or nutrient-restricted diet. On the contrary, a VD should include a wide variety of foods: fruits, vegetables, whole grains, pseudocereals, legumes, soy derivatives (e.g., tofu, tempeh), oils, nuts, seeds, herbs and spices [2], which are the most nutrient-dense foods and, as they are at the base of the food chain, the poorest in persistent organic pollutants [3].

There are no reliable sources of vitamin B12 (cobalamin) in plants [4], so nutrition professionals must always stress the need for supplementation.

Many pediatricians still have concerns about raising children on a VD and put great emphasis on the risk of nutritional deficiencies during critical phases of growth such as during weaning [5,6,7,8].

Deficiencies are likely to occur when parents are not aware of the dietary sources—and the right amount—of essential nutrients, and/or when, in the first two years of life, special attention is not paid to limiting the consumption of fiber, which abounds in a VD. High-fiber foods may inhibit absorption of fats and minerals (especially iron and calcium), are bulky and soon suppress the appetite, limiting an adequate intake of energy and nutrients [9].

Reliable sources of vitamin B12, vitamin D, iron, zinc, folate, proteins, calcium and omega-3 are critical for children’s health and development [10]. Therefore, healthcare professionals unanimously recommend seeking professional advice to plan children’s diet and to check their health status periodically [5,6].

Concerns about the need for more follow-up visits and blood tests in vegan children, compared to non-vegan children, can arise and might contribute to a “medicalization” of vegan children and an increased healthcare cost. It is clear that the choice of a VD for offspring is not always easy and cheap. However, data on these issues are not yet available.

Table 1 was designed to provide primary care pediatricians (PCPs) with a summary of Position Papers, reviews and guidelines on VD during infancy and childhood. Several nutrition societies and literature reviews have concluded that well-planned vegetarian diets, including a VD, can meet all of an infant’s and child’s nutritional requirements and ensure normal growth [2,5,11,12,13,14].

PCPs may not be prepared to help parents in planning a balanced VD because the teaching of nutrition in medical schools is often not planned and experiences with VD are lacking. Moreover, a skeptical approach of the PCP [15] hinders successful patient-physician communication: many parents may prefer not to inform the PCP and to adhere to a VD following other communicative channels, including dietitians. In any case, the role of both PCPs and dietitians is crucial: the first for a comprehensive growth and development assessment, and the second for diet planning. It follows that their common action increases the effectiveness in the counseling for these parents.

### Aim of the Study

The study aimed to:

- uncover the difficulties (i.e., emotional barriers, differences in viewpoint, lack of support) perceived by parents in their relationship with PCPs regarding a VD. These findings may be useful for PCPs to improve the information exchange with parents;
- know the level of involvement of any nutrition professional (i.e., dietitian, nutritionist) at any stage during the planning of the infant/child’s diet;
- investigate the parents’ motivation and criteria in choosing a VD for their offspring, and the parents’ adherence to the main dietary recommendations for vegans;
- collect data on the major issues regarding the implementation of a VD in everyday life.

## 2. Materials and Methods

### 2.1. Study Design and Population

A national cross-sectional survey was conducted between July and September 2020. Parents of children following a VD were invited to complete an online questionnaire. The questionnaire was available for completion on the Microsoft Forms platform. The distribution of the questionnaire was carried out via vegan weaning/nutrition Facebook groups.

A brief explanation of the purpose of the study and assurance of the confidentiality of the data was given in all cases. 

Participation in the survey was voluntary and anonymity was guaranteed. Before completing the questionnaire, all subjects were asked to self-assign a 4-character alphanumeric string. Parents with more than one child following a VD were invited to complete the questionnaire once for each child. Parents who completed the questionnaire more than once had to use the same string: in this way, we were able to calculate the number of parents recruited.

No child was involved in the study, and no personal information was collected. Approval by the ethics committee was not required.

### 2.2. Questionnaire

A 35-item questionnaire was designed from scratch to collect exploratory information on different topics, organized in sections as follows:

- Section 1: *Sample Characteristics*. This section contained 6 questions to investigate who was completing the questionnaire (mother/father) and demographic variables such as age, place of origin, and educational level. Offspring’s sex and age were also assessed.
- Section 2: *Parents-Primary Care Pediatricians Interaction*. This section contained 11 questions with the purpose of understanding some key points: (1) Do parents tell their PCPs that they have adopted a VD for their children? If not, why? (2) How do parents perceive the PCPs’ attitude regarding a VD? (3) How do parents judge the quality of information provided by the PCPs about a VD?
- Section 3: *Nutrition Counseling and Dietary Features*. This section contained 12 questions. Parents were asked whether their children had been following a VD since weaning or had started later in life, and whether they had consulted any nutrition professional to plan the infant/child’s diet. The questions assessing the food groups regularly included in the children’s diet at home and the regular vitamin B12 supplementation were designed according to the vegan nutrition guidelines for mothers and children [11], published by a panel of experts belonging to the Scientific Society for Vegetarian Nutrition (SSNV).
- Section 4: *Daily life issues of a Vegan Diet*. This section contained 6 questions to assess problematic issues that parents and children may often face: difficulty in finding vegan meals out of home, availability of a vegan menu at school, criticism from family members/friends and social exclusion.

For each item, the respondents were asked to choose a predefined answer listed after a question or statement. 

All the questions are listed in the Appendix S1 (see Supplementary Materials).

### 2.3. Data Analysis

Categorical data were described by absolute and relative (%) frequency. Comparisons were performed only among qualitative variables and the association between variables was assessed using the chi square test. 

The tables presented are descriptive and the statistically significant results (of the comparisons) were expressed by *p*-value and indicated in the text. Significance was fixed at 0.05 and all analyses were performed with SPSS technology (version 27).

## 3. Results

### 3.1. Sample Haracteristics

We collected data from 176 Italian parents (165 mothers and 11 fathers). Twelve parents completed the survey twice, for both of their children, so the number of responses (n = 188) was greater than the number of respondents (n = 176).

The socio-demographic characteristics of the parents are reported in Table 2. Most of them (93.8%) were mothers. More than half of the participants were aged 30–39 years (56.8%), came from Northern Italy (58.5%) and had a high educational level (bachelor’s degree or above, 63.6%).

Table 2 also shows the demographic features of the offspring: 188 children (52.7% females and 47.3% males) were included in this study; 71.8% had been following a VD since weaning.

### 3.2. Parents-General Pediatricians’ Relationship

One hundred and twenty-nine children had a female PCP, and 59 children had a male PCP. Most of the children (64.9%) had a PCP aged over 50 years.

More than 60% (63.8%) of the parents had informed their PCPs about choosing a VD for their offspring (Table 3). Among those who had not (36.2%), the most frequent reason was that they did not consider it to be essential (29.4%). Moreover, 27.9% of them were afraid of being judged, and 27.9% were aware of the PCPs’ opposition to a VD, so they avoided the topic. Other reasons (13.3%) were that parents were waiting for the periodic medical checkup, or they were under the supervision of a nutrition professional in private practice.

About one third (34.1%) perceived a negative attitude of their PCPs, whereas more than one third (36.7%) of the PCPs, though not sharing the VD choice, were considered to be sympathetic towards the parents’ decision. Just under one third (29.2%) of the participants had a positive perception of the physician’s attitude, referring to it as “welcoming” and “reassuring”.

Female PCPs tended to be perceived as more reassuring than males, although there was no statistically significant difference (*p* = 0.058). 

The PCP’s age (>60 years) was associated with the perception of a positive attitude (*p* = 0.041). The child’s sex did not condition the PCP’s attitude. 

To the question: “Did your pediatrician want to know more about why you chose a VD?”, 87.8% of the parents who had informed the physician answered “No”. However, in 61.6% of the cases, PCPs wanted to be sure that the children’s diet was nutritionally adequate.

We found neither association between the quality of information provided by PCPs and the geographical area, nor between the quality of information and the PCPs’ age.

### 3.3. Reasons and Criteria in Choosing a Vegan Diet for Children

The decision to exclude animal foods from the children’s diet was shared by both parents in 77.1% of the cases, and was made for various reasons, with health benefits and ethics being the most common (75.5%). Almost 5.7% of the interviewed subjects chose a 100% plant-based diet because of its low environmental impact, and 16.5% were not able to identify a single main reason.

Almost all the parents declared that they had looked for data regarding the nutritional adequacy of a VD, before deciding to raise their offspring on this kind of diet. Healthcare professionals (e.g., dietitians, nutritionists, private pediatricians with nutrition training) and scientific websites were the most popular sources, consulted in 88.2% and 46.2% of the cases, respectively.

Among healthcare professionals, 71.2% of the parents sought a medical dietitian (a physician nutrition specialist), and 28.2% sought a nutritionist (enrolled in the order of biologists) or a dietitian (enrolled in the order of dietitians) for dietary counseling. In the participants’ opinion, these healthcare professionals provided excellent information in 86% of the cases, sufficient in 13% of the cases and insufficient in 1% of the cases.

A total of 23.4% of the parents reported obtaining information from social networks and friends as well. As “other sources” (6.4%), the parents reported books, documentaries, internet tutorials, training courses and a Master’s in Vegetarian Nutrition and Dietetics.

### 3.4. Food Groups, Vitamin B12 Supplementation and Purchase of Convenience Foods

Table 4 shows data regarding food groups regularly included in the children’s diet, vitamin B12 regular supplementation and purchase of convenience foods.

In our cohort, about 90% (or more) of the children received six food groups (fruits, vegetables, grains, protein foods, nuts and seeds as well as fats and oils) on a daily basis, plus an individual B12 supplement.

Regular vitamin B12 supplementation was associated with asking for nutrition counseling (*p* = 0.031).

About 66.4% of the parents never, or rarely, buy convenience and ready-to-eat foods.

The perceived cost of a VD was not greater than the cost of an omnivorous diet in 88.8% of the cases.

### 3.5. Daily Issues of a Vegan Diet

According to the parents’ answers, the school was the most unlikely place to find vegan meals for children (Table 5). As a matter of fact, about one third (33.5%) of children were not provided with vegan lunches at school. This percentage may be even higher because 43.1% of the children involved in this study were not of school age or never ate in the school refectory, so their parents did not know whether schools catered for vegan pupils.

No significant association between the offer of vegan meals at school and the geographical area was found. However, a trend was observed. Vegan meals were more frequently available in Northern (45.4%) and Central (42.3%) Italy schools than in Southern Italy (20.0%).

To the question: “Out of the home, if your child wanted to taste animal foods …”, 146 parents (82.9%) would have not forbidden their children to eat animal foods, while 30 parents (17%) would have.

Almost all the participants (98.2%) thought that the most important issue was providing their children with the adequate amount of energy and nutrients they need to grow up in a healthy way.

## 4. Discussion

To the best of our knowledge, this survey is the first investigation which explores, quite extensively, the relationship between vegan parents and their PCPs, involvement of professional personnel such as dietitians or nutritionists, parental management of the children’s diet and challenges regarding the implementation of a VD in everyday life.

In the last few years, the interest in vegan weaning and a VD in the developmental age has risen sharply. The sociodemographic characteristics of vegetarians (including vegans) have been investigated by some authors [22,23], suggesting that vegetarians tend to have higher educational levels and social economic status. According to our results, a VD is very attractive to young parents (56.8% of the participants were aged 30–39 years) who have received a high educational level (63.6% had a bachelor’s degree, at least). The decision to follow a VD may indeed be influenced by parents’ level of education, which may help to choose the VD for possible health and environmental advantages.

As nutrition has a great impact on growth and development, PCPs should be provided with as much information as possible by the parents about the children and their environment, including dietary habits. Unfortunately, a considerable percentage of parents involved in the present survey did not share the decision of excluding animal foods with their PCPs for various reasons. Similar results were published by Baldassarre et al. in a recent survey involving 360 Italian families: in 22.7% of the cases, parents adopted alternative eating habits (e.g., semi vegetarian/lacto-ovo-vegetarian/vegan) without informing the pediatrician [16].

As a matter of fact, a considerable number of parents felt the need to change their PCP due to a conflict over the VD, and in about one third of the cases, an “oppositional, judgmental, skeptical, dissuasive” approach was perceived by parents. This kind of approach jeopardizes the doctor-patient alliance and might lead parents to adopt alternative dietary patterns without medical supervision. As a result of the lack of surveillance, parents may expose children to a higher risk of severe nutritional deficiencies, whose clinical signs (e.g., hypotonia, fatigue, neurological deficits, weight loss, etc.) can be easily mistaken for other conditions, including metabolic diseases. Hence, lack of information about the diet can make a differential diagnosis more complex, delaying the recognition of nutritional deficiencies [15,24]. 

A thorough nutrition education is not routinely given during an undergraduate course and residency. Consequently, many PCPs may not feel confident in dealing with alternative feeding practices. Indeed, according to our data, about half of the parents who had sought the PCP’s help to plan the children’s diet, reported that the physician had provided insufficient instructions. 

Female PCPs tended to be perceived as more reassuring than males (*p* = 0.058). This result could be explained by the fact that children are mostly brought to PCPs by their mothers, and among women there is a tendency towards greater empathy.

In our cohort, parents referred greater reassurance from the oldest PCPs, although we would have expected the youngest practitioners to be more updated and broader-minded in supporting a VD diet during infancy and childhood. The association between the PCP’s seniority and the parents’ positive perception may depend on the fact that the experience in dealing with parents improves with time. 

In the last few years, awareness of the benefits of plant-based diets has risen among the community of physicians [25] and an increasing number of courses/lectures on alternative dietary regimens have been established. We can speculate that the future generations of pediatricians will be more confident in planning plant-based diets for their young patients [26].

Parents appeared knowledgeable about the fact that a healthy VD should regularly include six food groups: fruits, vegetables, grains, protein foods, fats and oils as well as nuts and seeds [11]. Dark green leafy vegetables are excellent sources of folate, iron, calcium, potassium and vitamins A, C and K [27]. Whole grains are typically high in iron, zinc, magnesium, selenium, B vitamins and fiber [27]. With a large variety of foods during the day, children can obtain adequate amounts of essential amino acids [28], the building blocks of proteins. In plant-foods, unsaturated fatty acids predominate. Fats are necessary for normal brain development [29] and are the main source of energy for satisfactory growth during infancy [30]. We must point that the “nuts and seeds group” (an important source of calories, unsaturated fats, proteins and minerals) was offered less to the children than the other groups. Considering that 84.5% of the children included in our study were aged between 6 months and 6 years, we can surmise that their parents were probably afraid of sudden allergic reactions and/or choking. 

With regards to allergic reactions, current evidence suggests that the introduction of allergenic foods (e.g., peanuts, tree nuts, eggs, milk, etc.) during weaning, rather than later in life, is linked with a low probability of developing food allergies [31]. Secondly, the risk of choking largely depends on the shape and consistency of food: blending the nuts (to make a flour or creamy butter) is a strategy for safe swallowing and facilitates the child in terms of low masticatory ability. Professional nutrition advice is extremely important for parents who introduce their children to solid foods. 

During weaning and possibly beyond, milk continues to be an important source of energy and nutrients (Table 6).

The North American Society for Pediatric Gastroenterology, Hepatology, and Nutrition (NASGHAN) highlights that, when breastfeeding is not possible, in the first year of life human milk can only be replaced by appropriate commercial infant formulas [33]. Formulas for infants are available from plants: the soy- and rice-based formulas are indicated for infants for whom milk-formula is not suitable (allergy, VD, etc.) [34]. In contrast, plant-based beverages, even if fortified, are not suitable for infants and children, given that a formulated milk should represent the main source of nutrition [35,36,37].

Micronutrients, which consist of vitamins and minerals, are essential for children’s growth and development. Some of them are critical not only among vegan subjects, but also in the general population. Iron deficiency is the most common nutrient deficiency worldwide [38], and vitamin D, calcium [39], vitamin B12 deficiency [40] has been increasing both in developed and emerging countries. 

As a regular oral vitamin B12 supplementation is mandatory for those who consume no animal foods [11], we assessed the type of supplementation in the children’s diet. Most of the participants used an individual B12 supplement, as recommended by the guidelines. Very few parents used multivitamins, and it must be noted that these products may be insufficient or even counterproductive for optimal absorption of vitamin B12. Indeed, cobalamin can be degraded in the presence of vitamin C and copper, forming inactive by-products [4].

Parents who do not give their children any form of vitamin B12 supplement should be aware that a protracted vitamin B12 deficiency during the first years of life may lead to permanent brain injury [41,42]. It is therefore recommended to begin the vitamin B12 supplementation along with the introduction of solid foods [11].

An assessment of any other dietary supplementation (e.g., omega-3 fatty acids, iron, calcium, etc.) was not made because a well-planned VD that regularly includes all the food groups mentioned above ensures adequate amounts of macronutrients and micronutrients.

Almost all the parents asserted that they looked at data on the nutritional adequacy of VD before opting to raise their offspring as vegans, and the most popular sources were healthcare professionals and scientific websites. This data reveals a high sense of responsibility and awareness among parents on the detrimental effects that a not well-planned VD might have on their offspring. However, about 20% of parents reported obtaining information from social networks and friends, which might not be a reliable source of information.

Whilst today it is quite simple to find information, recipes and products to embrace a VD, out of home vegan meals were hardly (or not at all) available in 79.2% of the cases.

The Italian guidelines for school catering establish that institutes must provide adequate substitutions for children who do not eat meat or other animal products [43]: despite this obligation, schools do not always cater for vegan pupils. The inequality between schools in Northern-Central and those in Southern Italy found in the present study should be investigated on a larger sample.

In our survey, only a small minority of children experienced social exclusion for being vegan. This finding suggests that children do not pay too much attention to what their peers eat, while the stigma attached to a VD is still strong among adults and adolescents [44], despite the number of vegans continuing to rise [1].

Most parents stated that if their children wanted to taste animal foods, they would not prevent their children from doing that. This positive approach is essential, considering recent concerns raised by some authors who have hypothesized an association between the parents’ obsession with controlling what their children eat and the risk of eating disorders [45].

Almost all the participants shared the idea that the most important aspect is to provide children with adequate calories and nutrients. This means that children should not be on calorie-restricted diets, and calories should come from adequate amounts of carbohydrates, fats and proteins on a daily basis [46].

The present study has some limitations. The small sample size limits the generalization of the results to a larger population. Furthermore, only parents’ opinions are reported: the knowledge and the attitude of PCPs towards a VD would deserve to be thoroughly evaluated by means of appropriately designed surveys.

Parents were recruited via vegan weaning/nutrition Facebook groups. This might represent a recruitment-bias: those parents might represent a limited subset with more radical ideas or with a particular attitude to sharing on websites their experience about adopting a VD for their children. Further, we found a high number of parents declaring that they had looked for data regarding the nutritional adequacy of VD before the decision to raise their children on a VD. This data might be another recruitment-bias: those parents might represent a limited subgroup with a more proactive attitude, having decided to subscribe to vegan weaning/nutrition Facebook groups.

On the whole, our survey revealed that most parents were young and had received a high educational level. These data might be influenced by the fact that the survey was distributed via social media, which are mostly, but not exclusively, used by younger and highly-educated people.

However, to the best of our knowledge, the present study is the largest survey carried out so far among Italian vegan families. Moreover, it is the first study that has quite extensively investigated the relationship between PCPs and vegan parents, and how these parents manage their offspring’s diet, both at home and outside.

## 5. Conclusions

This survey provides an overview of the reasons why parents decide to raise their children on a VD, and of the difficulties regarding the implementation of a VD in everyday life.

Further, the interaction between parents and PCPs has been explored from the point of view of the former. Many PCPs are perceived as skeptical and unprepared when asked about raising children on a VD. However, parents’ perceptions may not correspond to reality: further research among PCPs is needed in order to better assess their knowledge and way of facing difficulties. 

Meanwhile, considering the striking percentage of parents who did not inform their PCPs about the VD, and who received insufficient information from the physician, strategies for PCPs to improve both their approach with parents and children’s health could include: (1) Always asking about the child’s diet, (2) Using the guidelines to advise parents correctly [9,11].

On the other hand, from the survey parents appeared knowledgeable regarding the main dietary recommendations and showed high awareness of the importance of relying on nutrition professionals to guarantee an adequate nutrition for their offspring. An investigation of the adequacy of the diet actually offered to the children was beyond the scope of the present study, but it would be very interesting to carry out by means of longitudinal studies.

## Figures and Tables

**Table 1:** Position Paper, Guidelines and Reviews on VD during infancy and childhood.

Authors	P.P./G.L. *	Review	Year	Highlights
Baldassarre, ME et al. [16]		✔	2020	Pediatric supervision, possibly in cooperation with dietitians/nutritionists, is critical in cases of vegetarian/vegan children. Vegan weaning should be discouraged and is contraindicated for ex-preterm infants because consistent findings that support both safety and feasibility are still lacking, and serious damage (e.g., slow growth, rickets, irreversible cognitive deficits, etc.) can occur.
Müller, P [17]		✔	2020	There is no solid evidence that a VD started in early childhood confers lasting health benefits. Children must be provided with a well-planned, diversified diet. Inadequate supplies of energy, proteins, long-chain fatty acids, iron, zinc, vitamin D, iodine, calcium and vitamin B12 are injurious to young children’s health.
Redecilla Ferreiro, S et al. [18]	✔		2019	It is preferable to recommend an omnivorous diet or, at least, an ovo-lacto-vegetarian diet during infancy and early childhood. As long as the diet is balanced and guarantees normal growth and development, the pediatric follow-up of vegetarian/vegan children has no particular features.
Rudloff, S et al. [19]	✔		2019	Healthcare providers should pay attention to the intake and status of vitamin B12, iron, zinc, iodine, DHA, calcium, protein and calories. Pediatricians, if possible in cooperation with dietitians, should monitor physical development and dietary intakes of children following vegetarian or restrictive diets.
Baroni, L et al. [11]	✔		2018	A 100% plant-based diet that is well-planned and supplemented with vitamin B12, is suitable during pregnancy, lactation, infancy and childhood. Healthcare professionals should follow an evidence-based approach in regard to the issue of a VD, in order to advise their patients correctly.
Agnoli, C et al. [2]	✔	✔	2017	Well-planned vegetarian diets that include a wide variety of plant foods and a reliable source of vitamin B12, fully meet all the nutritional needs.
Fewtrell, M et al. [5]	✔	✔	2017	An appropriately supplemented VD can support normal growth and development. Regular medical and dietetic supervision is recommended to avoid nutritional deficiencies.
Schürmann, S et al. [20]		✔	2017	Health benefits and risks of present-day vegetarian diets (with respect to infants, children and adolescents) should be widely investigated. Studies in pediatric subjects on a VD are scarce. Carefully conducted prospective studies with omnivorous control groups are necessary.
Melina, V et al. [12]	✔		2016	Appropriately planned vegetarian diets can fulfill nutrient needs and help people prevent or manage chronic diseases. These diets are a healthy and viable option across all life stages, including pregnancy, lactation, infancy, childhood, adolescence and older adulthood.
Craig, WJ [13]		✔	2010	Appropriately planned vegetarian diets are healthful, nutritionally adequate, and can help people prevent or manage chronic diseases. Compared with lacto-ovo-vegetarians, vegans tend to be slimmer, have a lower risk of cardio-vascular diseases and have a lower incidence of stroke and diabetes mellitus. Poorly planned vegetarian diets can be deficient in vitamin B12, calcium, vitamin D, zinc, iron and long-chain ω-3 fatty acids.
Craig, WJ et al. [14]	✔		2009	Appropriately planned vegetarian diets are healthful, nutritionally adequate, and can help people prevent or manage chronic diseases. These diets are a healthy and viable option across all life stages, including pregnancy, lactation, infancy, childhood, and adolescence.
Messina, V and AR Mangels [10]		✔	2001	With wise food choices, a VD can be adequate for children of all ages.
Mangels, AR and V Messina [21]		✔	2001	A VD can be planned to be nutritionally complete and supportive of an infant’s growth. When working with vegan families, healthcare providers should consider: composition of breast milk from vegan women, appropriate breast milk substitutes, supplements, type and amount of dietary fats, and solid food introduction.

* P.P.: Position Paper; G.L.: Guideline.

**Table 2:** Demographics of parents and children.

Parent Demographics		n = 176
**Parent**	• Mother • Father	165 (93.8%)
		11 (6.2%)
**Age (years)**	• 20–29 • 30–39 • 40–49 • >50	36 (20.5%)
		100 (56.8%)
		37 (21%)
		3 (1.7%)
**Place of origin**	• Northern Italy • Central Italy • Southern Italy and islands	103 (58.5%)
		47 (26.7%)
		26 (14.8%)
**Education**	• Primary school • Middle school • High school or vocational school • Bachelor’s degree • Master’s degree/Doctoral degree, etc.	–
		6 (3.4%)
		58 (33%)
		83 (47.2%)
		29 (16.4%)
**Children Demographics**		**n = 188**
**Sex**	• Male • Female	89 (47.3%)
		99 (52.7%)
**Age**	• 6–24 months • 2–6 years • 6–10 years • >10 years	78 (41.4%)
		81 (43.1%)
		13 (7%)
		16 (8.5%)
**Following a VD** **since …**	• Weaning • 12–24 months • 2–6 years • 6–10 years • >10 years	135 (71.8%)
		16 (8.5%)
		21 (11.2%)
		7 (3.7%)
		9 (4.8%)

**Table 3:** Parents’ answers to questions exploring parent-pediatrician relationship regarding a VD.

Question *		n = 188
**Pediatrician’s sex**	• Female • Male	129 (68.6%)
		59 (31.4%)
**Pediatrician’s age (years)**	• 30–39 • 40–49 • 50–59 • >60	20 (10.6%)
		46 (24.5%)
		74 (39.4%)
		48 (25.5%)
**Have you told your pediatrician about the vegan choice?**	• Yes • No	120 (63.8%)
		68 (36.2%)
		**n = 68**
**If not, why?**	• Fear of being judged • Pediatrician’s opposition • Not essential • Other • No answer	19 (27.9%)
		19 (27.9%)
		20 (29.4%)
		9 (13.3%)
		1 (1.5%)
		**n = 120 ^1^**
**How would you describe the pediatrician’s attitude?**	• Against, judgmental • Skeptical, dissuasive • Unfavorable, but understanding • Welcoming, reassuring	16 (13.3%)
		25 (20.8%)
		44 (36.7%)
		35 (29.2%)
		**n = 85 ^2^**
**Pediatricians’ arguments against VD during childhood**	• Micronutrient deficiencies • Protein deficiency • Ethical and biological reasons • No answer	25 (29.4%)
		16 (18.8%)
		13 (15.3%)
		31 (36.5%)
		**n = 188**
**Have you ever changed your pediatrician?**	• Yes • No • No, but I felt the need to do it • No answer	13 (7%)
		121 (64.4%)
		40 (21.3%)
		14 (7.4%)
**Have you ever asked your pediatrician for information on a VD?**	• Yes • No	33 (17.5%)
		155 (82.5%)
**How was the quality of information?**	• Excellent • Sufficient • Insufficient	7 (21.2%)
		9 (27.3%)
		17 (51.5%)

^1^ Number of parents who had informed their PCPs about the vegan choice. ^2^ Number of parents who described the PCPs’ attitude as: against, judgmental/skeptical, dissuasive/unfavorable but understanding. * The complete 35-item questionnaire is available in Appendix S1.

**Table 4:** Parents’ answers to questions aimed at evaluating: Administration of food groups, vitamin B12 supplementation and convenience foods to their offspring.

Question *		n = 188
**Which food groups do you regularly offer to your child?**	• Fruits • Vegetables • Grains • Protein foods • Nuts and seeds • Fats and oils	177 (94.1%)
		179 (95.2%)
		181 (96.3%)
		181 (96.3%)
		166 (88.2%)
		175 (93%)
**Does your child regularly take a vitamin B12 supplement?**	• Yes (individual supplement) • Yes (multivitamin) • No	164 (87.2%)
		12 (6.4%)
		12 (6.4%)
**How often do you buy convenience and ready-to-eat foods (e.g., burgers, cutlets, etc.) for your child?**	• Often (2–3 times/week) • Occasionally (2–3 times/month) • Rarely (once a month) • Never	12 (6.3%)
		51 (27.1%)
		61 (32.4%)
		64 (34%)
**In your opinion, is a VD more expensive than an omnivorous diet?**	• Yes • No	21 (11.2%)
		167 (88.8%)

* The complete 35-item questionnaire is available in Appendix S1.

**Table 5:** Parents’ answers to questions aimed at evaluating some issues encountered in implementing a VD for their children in everyday life.

Question *		n = 188
**What is the most unlikely place to find vegan meals?**	• School • Restaurant, bar … • Family and friends’ houses • None • Other	62 (33%)
		34 (18%)
		46 (24.5%)
		39 (21%)
		7 (3.7%)
**Does your child’s school cater for vegan pupils?**	• Yes • No • I do not know ^1^	**44 (23.4%)**
		**63 (33.5%)**
		**81 (43.1%)**
**Has your child ever been socially excluded?**	• Yes • No • No answer	7 (3.7%)
		173 (92%)
		8 (4.3%)
**Have you ever been criticized (by friends/family/teachers) because of the vegan choice?**	• No • Yes – Ethical and biological reasons – Protein deficiency – Micronutrient deficiency – Other – No answer	69 (36.7%)
		119 (63.3%)
		72 (60.5%)
		20 (16.8%)
		12 (10.1%)
		11 (9.2%)
		4 (3.4%)

^1^ Children were not of school age or never ate in the school cafeteria. * The complete 35-item questionnaire is available in Appendix S1.

**Table 6:** Feeding time: recommendations and plant-based alternatives to human (or cow) milk depending on child’s age and development.

Child’s Age.	Recommendation	Alternative
**0–6 months**	Exclusive breastfeeding [32]	Soy-based or rice-based infant formulas (type 1) [10,33].
**6–12 months**	Breastfeeding + solid foods [32]	Soy-based or rice-based infant formulas (type 2) + solid foods [10,33].
**From 12 months**	Breastfeeding + solid foods [32]	(1) Weaning is completed—solids are the main source of nutrition: plant-based (preferably fortified) beverages are suitable [10]. (2) Weaning is not completed—milk is the main source of nutrition: plant-based beverages are unsuitable. Soy-based or rice-based infant formulas (type 3) are needed [10].

## Supplementary Materials

The following is available online at https://www.mdpi.com/article/10.3390/nu13061796/s1. Appendix S1: The 35-item questionnaire.

## Abbreviations

VD: vegan diet; PCP: primary care pediatrician.
