# Supplementary material for: Raising Children on a Vegan Diet: Parents’ Opinion on Problems in Everyday Life
Source: Nutrients. 2021 May 25;13(6):1796. doi: 10.3390/nu13061796 (PMC8226937; doi:10.3390/nu13061796)
Supplement: Supplementary file 1 [file nutrients-13-01796-s001.zip › nutrients-1195216-supplementary.pdf]

# Appendix S1

## SECTION 1: SAMPLE CHARACTERISTICS

**1. Who is completing the questionnaire? ***
- Mother
- Father

**2. Your age group: ***
- 20-29 years
- 30-39 years
- 40-49 years
- > 50 years

**3. Place of origin: ***
- North (Emilia Romagna, Veneto, Friuli Venezia-Giulia, Liguria, Lombardy, Piedmont, Aosta Valley, Trentino Alto-Adige)
- Center (Tuscany, Umbria, Lazio, Marche, Abruzzo)
- South and Islands (Basilicata, Campania, Calabria, Molise, Apulia, Sicily, Sardinia)

**4. Educational level: ***
- Primary school
- Middle school
- High/professional school
- Bachelor’s degree
- Master’s degree/Doctoral degree, etc.

**5. Child’s sex: ***
- Male
- Female

**6. Child’s age group: ***
- 6-24 months
- 2-6 years
- 6-10 years
- > 10 years

## SECTION 2: PARENTS-PEDIATRICIANS’ RELATIONSHIP

**7. Your pediatrician’s sex: ***
- Male
- Female

**8. Your pediatrician’s age group: ***
- 30-39 years
- 40-49 years
- 50-59 years
- > 60 years

**9. Have you told your pediatrician that your child follows a vegan diet? ***
- Yes
- No

**10. If not, why?**

- o I am afraid of being judged
- o My pediatrician advises against a vegan diet in infants/children
- o I do not consider it essential
- o Other

**11. If yes, according to your own experience, how would you describe the pediatrician's attitude with regards to a vegan diet during infancy/childhood?**

- o Against, judgmental
- o Skeptical, dissuasive
- o Unfavorable, but understanding
- o Welcoming, reassuring

**12. If you answered "against, judgmental", or "skeptical, dissuasive", or "unfavorable but understanding", what was the main argument that your pediatrician put forward against a vegan diet?**

- o Human beings evolved to be omnivorous
- o It is not fair to impose a vegan diet on children
- o Protein deficiency
- o Mineral (calcium, iron, zinc…) and vitamin (B12, D) deficiency
- o Other

**13. Have you ever changed your pediatrician due to a conflict over the vegan diet?**

- o Yes
- o No
- o No, but I have seriously considered doing it

**14. Did your pediatrician want to know the reasons why you chose a vegan diet?**

- o Yes
- o No

**15. Did your pediatrician make sure that the child's diet was nutritionally adequate?**
*(They asked you about dietary supplements, follow-up with dietitians, etc.)*

- o Yes
- o No

**16. Have you ever asked your pediatrician for information on how to plan a vegan diet for your child? ***

- o Yes
- o No

**17. If yes, how was the quality of the information, in your opinion?**

- o Excellent
- o Sufficient
- o Insufficient

**SECTION 3: NUTRITION COUNSELING AND FEATURES OF THE DIET**

**18. Your child has been following a vegan diet since the time/age of… ***

- Weaning
- 12-24 months
- 2-6 years
- 6-10 years
- > 10 years

**19. The choice of excluding any animal foods from the child's diet was… ***

- Shared by both parents
- Made by one parent alone

**20. What is the main reason why you chose a vegan diet for your child? ***

- Respect for animals
- Health benefits
- Environmental impact
- Other

**21. Before making this choice, did you look for information regarding the adequacy and correct planning of a vegan diet during infancy/childhood? ***

- Yes
- No

**22. What type of sources did you consult?**
*(You can tick more than one option)*

- Friends, social networks (e.g. Facebook groups, Instagram, blogs…)
- Scientific websites (e.g. Pubmed, scienzavegetariana.it, Google Scholar…)
- Plant-based expert pediatricians, nutritionists, dietitians
- Other

**23. If you relied on a nutrition professional, they were a:**

- Medical dietitian
- Nutritionist
- Dietitian
- Other

**24. In your opinion, how was the quality of the information provided by your nutrition professional?**

- Excellent
- Decent
- Insufficient

**25. Which food groups do you regularly offer to your child? ***
*(You can tick more than one option)*

- Fruit
- Vegetable
- Grains
- Protein foods (e.g. legumes, soy and its derivatives, yogurt, etc.)
- Nuts and seeds
- Oils and fats

**26. Does your child regularly take a vitamin B12 supplement? ***

*(Individual supplement: containing only vitamin B12)*

- o Yes, an individual supplement
- o Yes, a multivitamin supplement
- o No

**27. In your opinion, is a vegan diet more expensive than an omnivorous diet? ***

- o Yes
- o No

**28. How often do you offer to your child packaged, convenience foods (e.g. burgers, cutlets) at lunch/dinner? ***

- o Often (at least 2-3 times/week)
- o Occasionally (2-3 times/month)
- o Rarely (once a month)
- o Never

**29. In your opinion, what is the most important issue in the children's diet?**

- o Providing them with "special" foods (e.g. tofu, seitan, tahini…)
- o Always providing them with adequate amounts of energy and nutrients
- o Protecting them from animal foods
- o Other

## SECTION 4: CHALLENGES OF A VEGAN DIET

**30. What is the most unlikely place to find vegan meals for your child? ***

- o School
- o Restaurant, bar…
- o Friends and family's houses
- o None
- o Other

**31. Does your child's school cater for vegan pupils?**

- o Yes
- o No
- o I do not know

**32. Have you ever been criticized (by friends/family/teachers) because of your vegan choice? ***

- o Yes
- o No

**33. If yes, what was the main argument they put forward against a vegan diet during infancy/childhood?**

- o Human beings evolved to be omnivorous
- o It is not fair to impose a vegan diet on children
- o Protein deficiency
- o Mineral and vitamin deficiency
- o Other

**34. Has your child ever been socially excluded by their peers because of the vegan diet? ***

- o Yes
- o No

**35. Out of the home, if your child wanted to taste animal foods… ***

- I would forbid it
- I would not forbid it

*** Question is mandatory.**
